# Risk factors for malalignment after intramedullary nail treatment of distal tibia fractures with associated fibula fractures

**DOI:** 10.1007/s00590-024-04062-x

**Published:** 2024-08-13

**Authors:** Yash P. Chaudhry, Jose M. Gutierrez-Naranjo, Micheal Raad, Diane Ghanem, Luis M. Salazar, Jason T. Goodrum, Kitchai Luksameearunothai, Boris A. Zelle, Erik A. Hasenboehler

**Affiliations:** 1 Department of Orthopaedic Surgery, Philadelphia College of Osteopathic Medicine, 4190 City Avenue, Philadelphia, PA 19131 USA; 2 Department of Orthopaedics, UT Health San Antonio, San Antonio, TX USA; 3 Department of Orthopaedic Surgery, The Johns Hopkins University School of Medicine, Baltimore, MD USA; 4 Faculty of Medicine Vajira Hospital, Navamindradhiraj University, Bangkok, Thailand; 5 Holy Spirit Medical Center Penn State Health, Orthopaedic Institute of Pennsylvania, Camp Hill, PA USA

**Keywords:** Tibial shaft fracture, Fibular fixation, Malalignment, Multiplanar interlocking screw fixation

## Abstract

**Purpose:**

Malalignment of distal tibia fractures can lead to malunion/nonunion or alter the limb mechanical axis which may cause arthritis. Proposed methods to decrease malalignment include fibular fixation or multiplanar interlocking screws, however these remain controversial. This study aimed to identify factors associated with malalignment in distal tibial fractures with associated fibular shaft fractures.

**Methods:**

A retrospective review was performed of distal tibia fractures with associated fibular shaft fractures treated with intramedullary nailing at two level one trauma centers between 2015 and 2019. Cases involving malalignment (> 5° of deviation from anatomic axis on either coronal/sagittal axis) on final follow-up (minimum three months postoperatively) were compared to those without malalignment with regard to demographics, fracture characteristics, intraoperative characteristics, and complications.

**Results:**

The rate of malalignment was 13%. On multivariate analysis, multiplanar distal interlocking screw fixation (odds ratio [OR], 0.18; 95% confidence interval [CI] 0.03–0.92) was associated with a decreased rate of final malalignment, while nail diameter > 10 mm was associated with a higher rate (OR, 4.05; 95% CI 1.25–13.11). Fibular fixation was not associated with malalignment.

**Conclusion:**

Multiplanar distal interlocking screws may protect against malalignment. Fibula fixation does not appear associated with a decreased rate of malalignment in distal tibia fractures treated with intramedullary nails.

**Level of Evidence:**

III.

## Introduction

Distal tibial shaft fractures are common fractures in the adult population that can be associated with substantial soft tissue injuries, bone loss, compartment syndrome, and associated fibular fractures [1]. Unstable fractures are typically treated with intramedullary nail fixation as this offers minimal soft tissue dissection and a load-sharing construct compared to conventional open reduction internal fixation [2, 3]. The treatment of combined distal tibial and fibula shaft fractures with intramedullary nail fixation, however, is frequently complicated by postoperative malalignment and/or malunion [1, 4]. A variety of described methods such as suprapatellar nailing in the semi-extended position, use of blocking screws, fixation of the fibula, and several indirect and direct reduction techniques are used to prevent malalignment [5–7]. Additional techniques to improve distal stability and prevent malalignment include distal interlocking screw fixation in more than one plane and adjunctive fixation of the fibula in cases involving associated distal fibula fractures [8–10]. This is particularly important as malalignment in the tibia may potentially lead to significantly higher rates of pain or early-onset arthritis in the knee or the ankle [11].

Given these controversies and the limited data available in the literature, the primary aim of this study was to assess the perioperative factors associated with malalignment in distal tibia with fibula fractures treated with tibial intramedullary nailing. Secondary outcomes included wound complications, infection, nonunion, or symptomatic hardware requiring an additional procedure for removal. We hypothesize that with the use of modern intramedullary implants and appropriate utilization of interlocking options, fibular fixation is not associated with any benefit with regard to rates of malalignment in distal tibia fractures.

## Material and methods

### Patient population

This retrospective cohort study included all patients who underwent intramedullary nailing for a distal tibia fracture with associated fibula fracture between 2015 and 2019 at two different level 1 trauma centers. Patients 18 years of age and older undergoing intramedullary nailing of extra-articular distal tibia fracture with associated fibula fracture (at the same level or distal), and with a minimum of 3 months of follow-up were included in this study. Fracture classification was according to the Arbeitsgemeinschaft für Osteosynthesefragen [AO]/Orthopedic Trauma Association [OTA] Classification and included 42-A, 42-B, 42-C, and 43-A. Extra-articular fracture was defined as distal to the isthmus of the diaphysis and extending through the flare of the distal tibia. Cases involving previous injury to the tibia and/or fibula, segmental tibia fractures, and those with incomplete follow-up, inadequate radiographs, or incomplete data were excluded. Cases involving a lateral malleolus fracture distal to the level of the tibial fracture were not addressed surgically if considered to be distant enough from the ankle joint and not to provide additional ankle stability. An intraoperative radiographic stress examination was performed after placement of the tibial nail. No intraoperative instability was found. Institutional review board approval was obtained from both medical centers involved in the study prior to the initiation of data collection.

### Data collection

A retrospective chart review was performed to collect the following data: patient demographics, fracture characteristics (AO/OTA classification, open versus closed fracture, location of fibula fracture, presence of malleolar fracture), operative technique (presence of fibula fixation, number of interlocking screws, configuration of interlocking screws [single plane such as two medial to lateral (ML) screws or multiplanar such as two ML screws and one anterior to posterior (AP) screw], blocking screws, and approach), preoperative and postoperative alignment at final follow-up, wound complications or infection, nonunion, and symptomatic hardware requiring removal.

### Outcomes

The primary outcome was the presence of malalignment. On full-length tibia radiographs, we measured alignment by using a line drawn down the center of the anatomic axis of the tibia shaft and a line perpendicular to the distal tibia articular surface. Coronal malalignment was defined as the angle obtained from the cross section of these two lines on anterior/posterior (AP) radiographs ≥ 5°. Full-length lateral tibia radiographs were used to determine sagittal malalignment, defined as angulation of ≥ 5° between a line drawn down the center of the anatomic axis of the tibia shaft and a line perpendicular to the distal tibia articular surface. These definitions for coronal and sagittal malalignment were chosen based on prior literature on the topic [12]. Alignment was measured both on immediate postoperative radiographs and radiographs at last follow-up date. Final malalignment was defined using the above at the final follow-up.

### Statistical analysis

The study sample was divided into two cohorts, those with associated fibular fracture fixation and those without. These cohorts were compared with respect to patient demographics, fracture characteristics, intraoperative factors, and postoperative outcomes. An additional comparison of these variables was performed between cases involving the development of final malalignment. Continuous variables were reported as mean (standard deviation [SD]) and compared using student’s t-test. Categorical variables were reported as frequency (percentage) and compared using Pearson’s Chi-squared test or Fisher’s exact test, as appropriate. A multivariate logistic regression was used to identify independent risk factors for final malalignment. Results of the regression model were reported as odds ratios (OR) with 95% confidence intervals (95% CI). Statistical significance was set at *p* < 0.05. All statistical analysis was performed using JMP 17.0 (SAS Institute, Cary, NC).

## Results

### Patient characteristics

One hundred and twenty-two patients were included in this study. Mean age at the time of injury was 44 years (SD 18), and 35% (*N* = 43) of patients were female (Table 1). Thirteen percent (*N* = 17) of the patients in this study underwent concomitant fibular fixation (four with intramedullary screws, nine with plate fixation). Sixteen (13%) patients had malalignment on final follow-up; of these sixteen patients, five (31%) had evident varus malalignment on immediate postoperative radiographs, while the remaining eleven (69%) patients developed malalignment after surgery.

**Table 1:** Perioperative characteristics of patients treated surgically with intramedullary nailing of distal tibia fractures with associated fibula fractures comparing fibular fixation to no fibular fixation

Variable	Fibular fixation (*n* = 17)	No fibular fixation (*n *= 105)	Total (*n* = 122)	*p*-value
*Preoperative factors*				
Age (years)	50 ± 20	43 ± 17	44 ± 18	0.272
Female	8 (47%)	35 (33%)	43 (34%)	0.212
Open fracture	6 (35%)	39 (37%)	45 (37%)	0.884
Associated malleolar fracture	8 (47%)	23 (22%)	31 (25%)	0.027
*AO/OTA fracture type*				
42-A1	4 (24%)	35 (33%)	39 (32%)	0.219
42-A2	4 (24%)	28 (27%)	32 (26%)	
42-A3	2 (12%)	15 (14%)	17 (14%)	
42-B1	2 (12%)	4 (3.8%)	6 (4.9%)	
42-B2	3 (18%)	10 (9.5%)	13 (11%)	
42-B3	0 (0%)	8 (7.6%)	8 (6.6%)	
42-C1	0 (0%)	1 (1.0%)	1 (0.8%)	
42-C2	0 (0%)	2 (1.9%)	2 (1.6%)	
43-A1	0 (0%)	1 (1.0%)	1 (0.8%)	
43-A2	1 (5.9%)	0 (0%)	1 (0.8%)	
43-A3	1 (5.9%)	1 (1.0%)	2 (1.6%)	
*Fibular fracture level*				
Proximal	0 (0%)	35 (33%)	35 (29%)	< 0.001
Same level	4 (24%)	47 (45%)	51 (42%)	
Distal	11 (65%)	19 (18%)	30 (25%)	
Segmental	2 (12%)	4 (3.8%)	6 (4.9%)	
*Intraoperative factors*				
Infrapatellar approach	4 (24%)	24 (23%)	28 (23%)	1.000
Nail diameter (mm)	10 ± 0.8	10 ± 0.9	10 ± 0.9	0.501
Nail diameter > 10 mm	6 (35%)	22 (21%)	28 (23%)	0.200
Blocking screw use	3 (18%)	3 (2.9%)	6 (4.9%)	0.035
Number of proximal interlockers	1.9 ± 0.3	1.7 ± 0.5	1.7 ± 0.5	0.084
Number of distal interlockers	2.2 ± 0.5	2.4 ± 0.5	2.3 ± 0.5	0.387
*Distal screw planes*				
Uniplanar	4 (24%)	64 (61%)	68 (56%)	0.007
Multiplanar	13 (76%)	41 (39%)	54 (44%)	
*Postoperative variables*				
Time to follow-up (months)	12 ± 8.5	9.7 ± 8.4	10 ± 8.4	0.247
Wound complication/infection	2 (12%)	12 (11%)	14 (11%)	1.000
Malunion/nonunion	1 (5.9%)	9 (8.6%)	10 (8.2%)	1.000
Symptomatic hardware (Requiring removal)	0 (0%)	3 (2.9%)	3 (2.5%)	1.000
Final malalignment	1 (5.9%)	15 (14%)	16 (13%)	0.466

*AO/OTA* Arbeitsgemeinschaft für osteosynthesefragen/orthopedic trauma association

### Univariate analysis

Within the cohort involving fibular fixation, there was a higher rate of associated malleolar fracture (*p* = 0.027) and fibular fracture level distal to the tibial fracture (*p* < 0.001). No other differences between preoperative characteristics or intraoperative fractures were observed. Additionally, there were no differences in rates of wound complication/infection (*p* = 1.00), malunion/nonunion (*p* = 1.00), or symptomatic hardware requiring removal (*p* = 1.00). A higher rate of final malalignment was observed in cases with nails diameter > 10 mm (47% vs. 20%, *p* = 0.021) (Table 2). There was no association between malalignment and the use of blocking screws (*p* = 0.578). Four cases with final malalignment (25%) involved multiplanar distal interlocking screw fixation, compared to 50 (47%) of the cases without final malalignment (*p* = 0.096).

**Table 2:** Perioperative characteristics of patients treated surgically with intramedullary nailing of distal tibia fractures with associated fibula fractures comparing those with malalignment at final follow-up versus those without malalignment

Variable	Malalignment at last follow-up (*n* = 16)	No malalignment at last follow-up (*n* = 106)	Total (*n* = 122)	*p*-value
*Preoperative factors*				
Age (years)	47 ± 16	43 ± 18	44 ± 18	0.433
Female	9 (56%)	34 (32%)	43 (35%)	0.059
Open fracture	6 (38%)	39 (37%)	45 (37%)	0.956
Associated malleolar fracture	3 (19%)	28 (26%)	31 (25%)	0.759
*AO/OTA fracture type*				
42-A1	5 (31%)	34 (32%)	39 (32%)	0.721
42-A2	5 (31%)	27 (25%)	32 (25%)	
42-A3	1 (6.3%)	16 (15%)	17 (14%)	
42-B1	1 (6.3%)	5 (4.7%)	6 (4.9%)	
42-B2	1 (6.3%)	12 (11%)	13 (11%)	
42-B3	3 (19%)	5 (4.7%)	8 (6.6%)	
42-C1	0 (0%)	1 (0.9%)	1 (0.8%)	
42-C2	0 (0%)	2 (1.9%)	2 (1.6%)	
43-A1	0 (0%)	1 (0.9%)	1 (0.8%)	
43-A2	0 (0%)	1 (0.9%)	1 (0.8%)	
43-A3	0 (0%)	2 (1.9%)	2 (1.6%)	
*Fibular fracture level*				
Proximal	6 (38%)	29 (27%)	35 (29%)	0.523
Same level	8 (50%)	43 (41%)	51 (42%)	
Distal	2 (13%)	28 (26%)	30 (25%)	
Segmental	0 (0%)	6 (5.7%)	6 (4.9%)	
*Intraoperative factors*				
Infrapatellar approach	5 (31%)	23 (22%)	28 (23%)	0.397
Nail diameter (mm)	10 ± 1.3	10 ± 0.8	10 ± 0.9	0.344
Nail diameter > 10 mm	7 (47%)	21 (20%)	28 (23%)	0.021
Blocking screw use	1 (6.3)	5 (4.7%)	6 (4.9%)	0.578
Number of proximal interlockers	1.8 ± 0.4	1.7 ± 0.5	1.7 ± 0.5	0.448
Number of distal interlockers	2.1 ± 0.5	2.3 ± 0.5	2.3 ± 0.5	0.284
*Distal screw planes*				
Uniplanar	12 (75%)	56 (53%)	68 (56%)	0.096
Multiplanar	4 (25%)	50 (47%)	54 (44%)	
Fibular fixation	1 (6.3%)	16 (15%)	17 (14%)	0.466
*Postoperative variables*				
Time to follow-up (months)	13 ± 7.5	9.7 ± 8.5	10 ± 8.4	0.106

*AO/OTA* Arbeitsgemeinschaft für osteosynthesefragen/orthopedic trauma association

### Multivariate analysis

The variables of open/closed fracture, approach, nail diameter, blocking screw use, fibular fixation, and distal interlocking screw configuration were included in the regression model. Multiplanar distal interlocking screw fixation was associated with a decreased risk of final malalignment (OR, 0.18; 95% CI 0.03–0.92). Nail diameter > 10 mm was associated with an increased risk of final malalignment (OR 4.05; 95% CI 1.25–13.11) (Table 3).

**Table 3:** Multivariate analysis of risk factors for final malalignment in distal tibia fractures with associated fibula fractures treated with intramedullary nailing

Variable	Odds ratio	Upper 95% CI	Lower 95% CI	*p*-value
Open fracture	1.17	0.36	3.86	0.794
Infrapatellar approach	1.03	0.26	3.97	0.971
Nail > 10 mm	4.91	1.42	17.0	0.012
Blocking screw	4.26	0.26	68.57	0.307
Fibular fixation	0.47	0.04	5.14	0.537
Multiplanar distal interlocking screws	0.18	0.03	0.92	0.040

*CI* Confidence interval

## Discussion

The aim of this study was to assess the factors associated with malalignment in distal tibia fractures treated with intramedullary nailing. The results of this study suggest that multiplanar distal interlocking screw fixation may be protective against the rate of malalignment, but that fibular fixation may not provide any benefit. Finally, a nail diameter greater than 10 mm was associated with an increased risk of malalignment, likely representing either poor bone quality or insufficient intramedullary filling.

While the use of two distal interlocking screws has been demonstrated to have superior outcomes for distal third tibial shaft fractures in comparison to a single distal interlocking screw [13], the optimal configuration of screw placement remains controversial. The metaphyseal flare and widening of the intramedullary canal as well as the decreased purchase of interlocking screws in metaphyseal bone leaves fractures in the distal tibia vulnerable to increased instability in the presence of intramedullary fixation devices [14]. In theory, the use of multiple perpendicular or oblique distal interlocking screws would lead to better maintenance of reduction by providing stability in multiple planes. A biomechanical cadaveric study conducted by Attal et. al. in 2014 found increased stability for multidirectional screw configuration (less rotation and translation at fracture site) when comparing conventional distal interlocking (two medio-lateral [ML] screws) in reamed intramedullary nailing of simulated distal tibia fractures [15]. Additionally, they reported that while the use of fibular plating increased stability when using the conventional cohort, it did not confer any increased stability in the multidirectional cohort [15]. However, similar cadaveric biomechanical studies conducted by Xavier et. al. and Lucas et. al. comparing multiple distal interlocking configurations (both uniplanar and multiplanar, two versus three screws) found that the 2 ML distal interlocker screw configuration was non-inferior to multiplanar configurations (with two or three screws) with regard to load carrying capacity and stability in torsion, compression, and bending tests [16, 17]. These studies remain limited, as compared to studies in human subjects, which are able to account for the biologic factors and bone healing. Recent years have seen the introduction of angle stable interlocking screws (encased in a polyetheretherketone sleeve) to tibial intramedullary nails to reduce toggle of the screws within the nail and potential migration of the interlocking screws [18], however, these advanced screw fixations were not used in our study.

The literature on the effectiveness of fibula fixation in distal tibia fractures remains controversial. In a study of 120 extra-articular and simple intra-articular distal tibia fractures with associated fibula fractures, van Veelen et. al. found a higher rate of angular malalignment and infections requiring revision surgery in cases involving fibular plate fixation [9].

Intramedullary fixation and plate fixation provide stability in different planes, thus adjunctive fibular fixation’s effect on malalignment may be different in each.

There may be a host of other factors that play a much larger role in distal tibia malalignment. For example, centralizing the guidewire is of extreme importance, particularly in meta-diaphyseal fractures in which the contact surface between the nail and the intramedullary canal is limited [12]. An eccentric position of the guidewire will lead to malreduction at the fracture site, irrespective of fibular fixation [12].

The only other factor the current study found to be associated with malreduction was a nail diameter > 10 mm. This may seem contradictory as larger nail size is thought to be associated with greater stability and maintenance of reduction [19] but is most likely a reflection of poor bone quality, thinner cortices and inability to provide a snug endosteal fit in a wide distal metaphysis [2]. Thus, increasing nail size likely does not add to stability in the same way it would for a fracture at the isthmus or diaphysis. Other studies have reported decreased rates of malalignment with the suprapatellar approach (particularly in semi-extended position) and the use of blocking screws [20, 21]. While the current study did not find either to have statistically significant relationships with malalignment, this may be explained by sample size as well as variability in surgeons.

In theory, a second incision for fixation of the fibula, especially for plate fixation in comparison to an intramedullary implant, would increase the risk of wound healing issues, possible infection, or need for hardware removal [22]. While this was demonstrated in a study by Prasad et. Al [23], we did not observe similar outcomes. Moreso, while some concerns exist over the risk of tibial nonunion with fixation of the fibula [24], this was also not demonstrated in the current study.

**Fig. 1:**
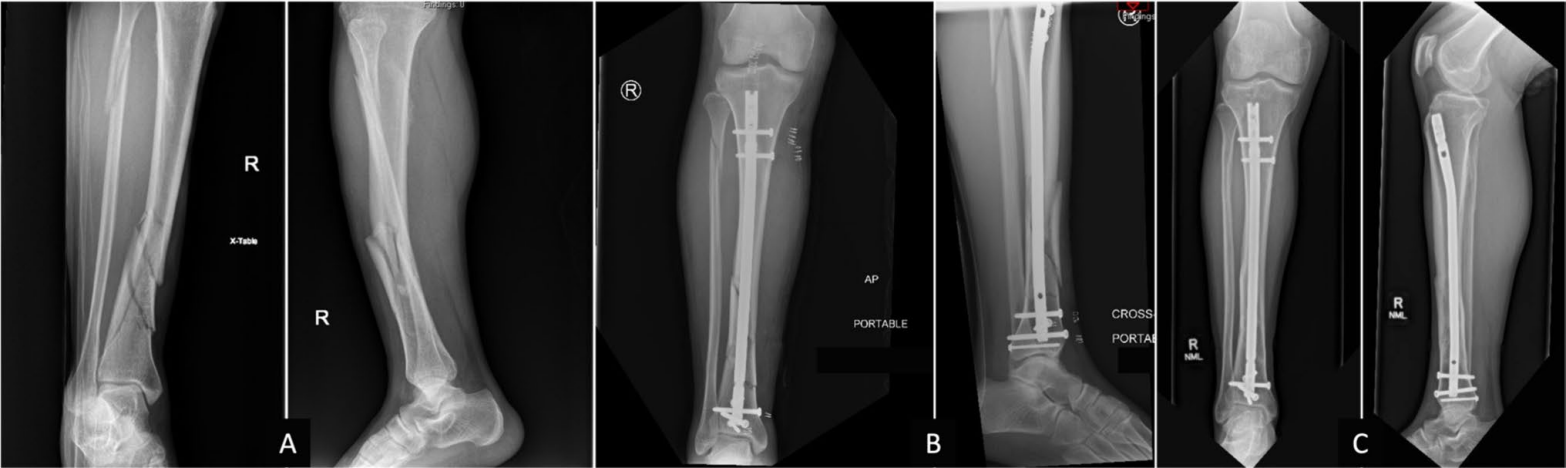
46 years old male with closed AO-type 42-C1 distal tibia fracture with associated proximal fibula fracture treated with intramedullary nailing. **A** Preoperative AP and lateral images, **B** immediate postoperative pictures, and **C** final follow-up pictures with no malalignment

**Fig. 2:**
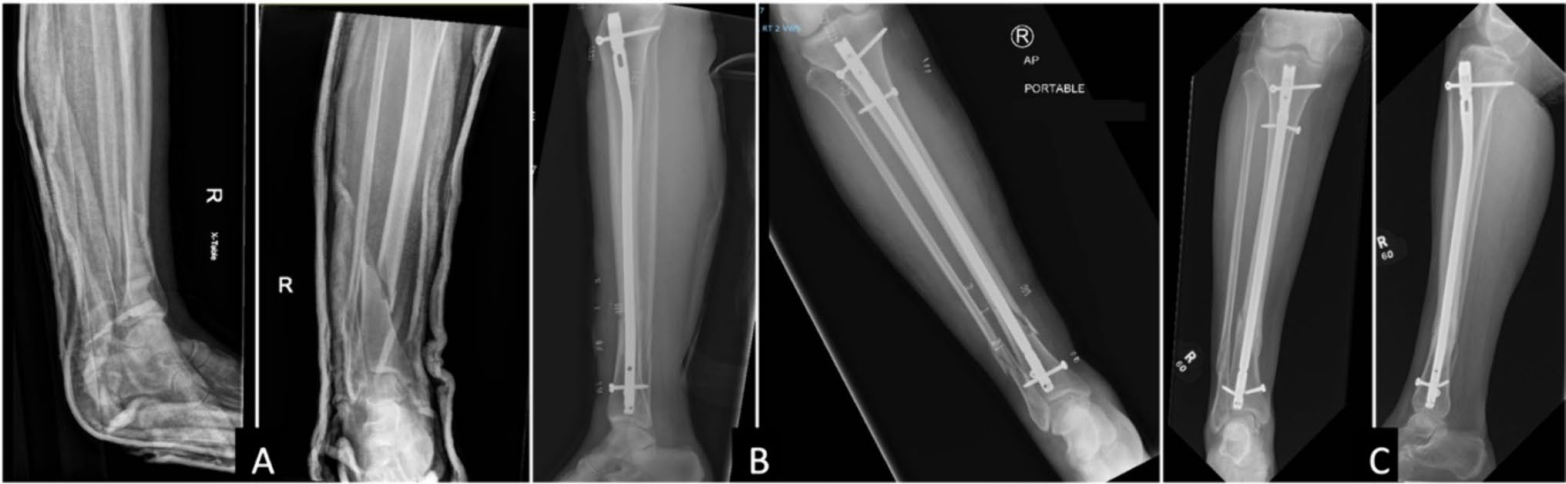
48 years old female with closed AO-type 42-B1 distal tibia fracture with associated fibula fracture treated with intramedullary nailing. **A** Preoperative AP and lateral images, **B** immediate postoperative pictures without malalignment, and **C** final follow-up at three years with varus malalignment and broken interlocking screws

**Fig. 3:**
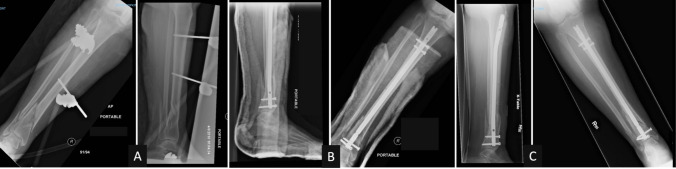
48 years old female with closed AO-type 42-A1distal tibia fracture with associated fibula fracture treated with intramedullary nailing. **A** Preoperative AP and lateral images, **B** immediate postoperative pictures with slight apex anterior malalignment, and **C** one-month follow-up demonstrating progressive apex anterior (sagittal) malalignment

Three examples of cases involved in this study are demonstrated in Figs. 1, 2, 3. Figure 1 displays the imaging of a 46 year-old male with an AO-type 42-C1 distal tibia fracture that was treated with a 10 mm nail through a suprapatellar approach with multiplanar distal interlocker fixation. The fibular fracture was not addressed. At final follow-up 39 months later, he maintained excellent alignment. Figure 2 demonstrates a case in which the fracture went on to malalignment at final follow-up. A 48 year-old female with an AO-type 42-B1 distal tibia fracture was treated with a 10mm nail through an infrapatellar approach with multiplanar interlocker fixation distally with the use of one ML and one AP interlocking screws. The fibula was not fixed in this case either. While the immediate postoperative radiographs did not meet the threshold for malalignment, at three years follow-up, the fracture was seen in varus malalignment with broken interlockers. Figure 3 demonstrates another case ending in malalignment, involving a 48 year-old female with a closed AO-type 42-A1 distal tibia fracture treated with an 11 mm nail with multiplanar interlocker fixation distally (one ML, one oblique). At her one-month follow-up, she was found to have progressed to sagittal malalignment.

This study has several limitations. As a retrospective cohort study, it is subject to potential selection bias. Rotational malalignment was not included for analysis since this cannot be measured with conventional imaging. Rotation was assessed during the clinical exam in follow-up, and because it was clinically irrelevant if found, it was not documented in degrees. Additionally, even with the popularity of the cutoff of 10° of malrotation commonly cited in orthopedic trauma literature [25–30], it has not been associated with any decrease in functional outcomes as reported by Theriault et. al.[29]. Finally, using the uninjured limb as a reference may not be a reliable way to assess rotation. Clementz et. al. assessed a random sample of 100 subjects without history of lower extremity injury and found a range of a difference of −11 to + 15 degrees between the two tibias [31]. Over a quarter of subjects had a natural difference over 6° as well. Similarly, the current study was unable to assess restoration of length as several of the postoperative and follow-up radiographs were taken in multiple shots (e.g., separate proximal and distal radiographs) which prohibited appropriate measurement of tibial length. The decision to use fibular fixation in each case was based on the discretion of the surgeon. While we attempted to control for that using multivariate analysis to account for different techniques such as approach and use of blocking screws, skill and comfort using each of these techniques also varies and could be a source of bias. Additionally, the sample size may have limited the ability to assess certain secondary outcomes such as wound complications and nonunion rates. Finally, longer follow-up could have allowed the evaluation of potential long-term morbidity of malalignment. Strengths of this study include its multicenter nature, including treatment from multiple surgeons and the use of several techniques. We were also able to present a fairly large sample size with an extended follow-up periods of at least three months, which is known to be difficult within trauma populations [32]. Further studies are warranted to determine the role of angle stable interlocking screws and how they may affect malalignment.

In conclusion, our results suggest that the use of multiplanar distal interlocking screw configurations may protect against malalignment. Additionally, modern nailing techniques and the utilization of contemporary intramedullary implants likely negate the need for concomitant fibula fixation. Fibula fixation should be limited to special situations, such as unstable ankle mortise, unacceptable displacement of the fibula, or as a reduction tool to achieve appropriate length.

## Data Availability

Available on request.
